# Social Acceptance of Greywater Reuse in Rural Areas

**DOI:** 10.1155/2022/6603348

**Published:** 2022-09-26

**Authors:** Issam A. Al-Khatib, Abed al Hamid U. Al Shami, Gonzalo Rodriguez Garcia, Ilke Celik

**Affiliations:** ^1^Institute of Environmental and Water Studies, Birzeit University, Birzeit, West Bank, State of Palestine; ^2^Universal Institute of Applied and Health Research, Nablus, State of Palestine; ^3^Department of Civil and Environmental Engineering, South Dakota School of Mines and Technology, 501 E St Joseph St, Rapid City, SD 57701, USA

## Abstract

Like many countries, Palestine suffers from water scarcity. Here, treated greywater is considered an essential nonconventional water resource. We aim to identify some wastewater reuse and disposal practices in rural areas and assess the acceptance level of different reuses of greywater. We conducted a survey analysis in four villages with a strong agricultural activity of the western Bethlehem Governorate. The level of acceptance of greywater reuse was generally independent of demographic variables like family size, income, or water bill, with a few exceptions regarding gender, age, and level of education. Centralized treatment was more valued than treatment at home, which presented similar acceptance levels than no treatment and might indicate a lack of trust in this alternative. The only reuse alternative trusted across treatments was bush irrigation (3.53-3.86 on a five-point Likert scale), but other options without clear, direct human contact like crop irrigation (3.14-3.62), stone cutting (3.19-3.36), and construction (3.12-3.42) also received considerable support. Reused perceived as having direct contact with humans was rejected, as it was the flushing of public toilets (2.59-2.7), aquaculture (1.98-2.37), olive pressing (1.85-1.94), and drinking (1.62-1.72). Relatively new reuse, car washing (2.95-3.17), was somewhere in between, partially because of its novelty. To increase this and other reuses, we strongly encourage local authorities to inform the population about the potentialities of greywater reuse.

## 1. Introduction

Nearly one-fifth of the world already exceeds its regional capacity for freshwater consumption, and its demand is increasing due to population growth and expanding industrial and agricultural sectors [1–6]. The discharge of pollutants to the aquatic ecosystem aggravates this problem. Domestic, industrial, and agricultural wastewater degrade freshwater bodies, further limiting existing resources [7–9]. Arid and semiarid regions are particularly susceptible to water scarcity. On the one hand, the introduction of technological innovations, namely, deep tubewells and high-powered pumps, have allowed for a continual, unsustainable drawdown of aquifers [10]. On the other, climate change is expected to cause a decline in precipitation and increased temperatures, implying higher evaporation rates. In addition, climate change could potentially increase existing regional tensions [10]. Increasing water demand is also critical in Palestine due to its limited water resources, caused by the region's arid climate and current water policies. Difficulties accessing local and underground resources have led Palestinians to search for unconventional water resources, like treated wastewater or harvested rainwater, even when the increasingly unpredictable seasons make the latter unstable [11–14]. People of the West Bank also have insufficient access to the sewage system. While the average is 30%, it varies widely between governorates: from 0% in Tubas to 59% in Qalqilya [15]. There are also significant differences between urban and rural communities, with 45.8 and 7.6% access, respectively [16]. Two-thirds of the population use cesspits, which are emptied by vacuum tankers. The latter dump their contents in the sewers and creeks, open areas, and dumpsites. The existing wastewater treatment plants (WWTPs) have not been specifically designed to treat the sludge collected from septic tanks. However, some plants accept these trucks, e.g., Al-Bireh WWTP [17]. This will cause a problem in the metals and potential phosphate removal. Also, it increases the large objects load to the plant. Since the late 1990's, Palestinian local nongovernmental organizations (NGOs)—financially supported by international aid agencies—have implemented about 600 nonconventional onsite treatment systems. Most of them separate greywater and blackwater, the former for garden irrigation and the latter disposed of in cesspits. Some of the greywater is subject to treatment before reuse, while the other is not subject to treatment before use. None of these solutions is wholly accepted or regarded as risk-free [18]. There are different technologies for wastewater treatment, for example, the use of constructed wetlands as a posttreatment step after an up-flow anaerobic sludge blanket (UASB) reactor is a promising technology for wastewater reclamation and reuse in arid and semiarid areas [19]. In addition, using of gravel filter followed by sand filter or horizontal flow sand filter for the treatment of greywater was found to be promising, simple, and low-cost technique [20].

Treated greywater—wastewater produced from households without toilet water but including water from bathtubs, showers, hand basins, laundry machines, and kitchen sinks—is another nonconventional water resource that can reduce water scarcity [14, 21–25]. It accounts for about 75% of the domestic wastewater, although it could be up to 100% if dry latrines are used [26, 27]. Blackwater—toilet water—represents a smaller fraction of the domestic wastewater and has higher nutrient content than COD-rich greywater [28]. Because of that, it can be beneficial to separate them at the source, leading not only to greywater reuse but also to possible blackwater and kitchen waste combined disposal [29]. While greywater separation and reuse have clear advantages, poor management could cause environmental or human health problems [30–32]. One example of a poor manager is poor storage, which might turn greywater septic in 1-2 days, causing odors and deteriorating quality [32]. Another is cross-contamination with blackwater. Even infractions as minor as 6-8% further treatment might be required [33].

The reuse of wastewater is a contested subject worldwide [34, 35]. For instance, Dolinčar and Saunders [36] reported that the acceptance of recycled water use varies with education, age, income, and gender. Acceptance also varies across the globe. Abdelrahman et al. [37] showed that the majority of the public (55%) in the United Arab Emirates agreed to use treated wastewater for irrigation of nonfood crops but refused to use it for irrigation of food crops (66%). In the Omanite Muscat Governorate, 76% of the population accepted reusing greywater for gardening, 66% for toilet flushing, and 53% for car washing [38]. In Turkey, respondents to a nationwide questionnaire were concerned with the health risks of reused greywater. Still, they accepted its reuse for applications without close personal contact like toilet flushing, cleaning roads, or construction [39].

Public dissemination campaigns are necessary if we want the general public to be (1) aware of the risks and benefits of greywater reuse and (2) supportive of it. When it comes to water reuse projects, it is necessary to work with the public from the beginning, mainly if drinking is one of the intended options [40]. In a review of greywater reuse, Radingoana et al. [41] indicated that public acceptance is as much a key factor for the success of these projects as the technical aspects. They also suggested that communicating best practices on treatment and reuse to local communities is one of the most important steps towards securing a sustainable water supply in rural areas. As necessary as supplying technical information in an accessible manner might be, however, water reuse also requires personal engagement on the part of the citizens. Campaigns with attitude-related messages—e.g., greywater reuse will protect water resources—and personal norm-related ones—treatment systems are the norm—were found to be more successful in this regard [42].

In the Palestinian territories, greywater represents about 80% of total domestic wastewater [43]. In rural areas, 90% of households separate greywater and use it without treatment in their home gardens, while blackwater is directed to the cesspit [44]. This use of untreated wastewater may cause environmental and health problems: polluting soil and groundwater, creating offensive smells, and transmitting waterborne diseases [45]. The reuse of greywater outside of households increased from 2009-2012. Most of the new governmental buildings included wastewater treatment plants and used the treated water for irrigation [46]. For example, in Bethlehem, Nablus, and Ramallah, the police car maintenance centers have two treatment plants each. One is for wastewater, and its treated effluent is used for irrigation [47]. The other treats car washing water, reusing its effluent for the same purpose [48, 49]. Similar technologies have also been implemented within public buildings in neighboring countries [50]. According to Al-Khatib et al. [51], although there exists a strategy about water reuse (Decree No.14, 2014), the guidelines on using treated wastewater remains missing in the Palestinian water law, thus hindering its implementation.

The acceptance of greywater reuse in the West Bank was assessed by Abu-Madi et al. [52] in the rural areas of the Ramallah and Al-Bireh Governorate. More recently, Thaher et al. [53] interviewed owners with greywater treatment plants at home. The latter found out that the primary reason for supporting greywater reuse was its irrigation potential, and to a lower extent, avoiding cesspit discharge and water scarcity. The most significant barriers for further implementation they identified included odor emission and insect infestation and lack of monitoring by the implementing agency. In the rural areas of the eight governorates covered, the average supporter of greywater treatment had a low education level and came from a large family with low income.

In this study, we aim to identify current wastewater practices in rural Palestine, namely, the western Bethlehem Governorate, which, to the best of our knowledge, had not been assessed before. Critical aspects of the study include understanding the greywater concept among the population, a fraction of households separating greywater from discharged sewerage, or the percentage of them using untreated greywater. More importantly, we want to assess the level of acceptance of treated greywater for different purposes and evaluate whether it varies depending on the treatment given or on the socioeconomic status of the household.

## 2. Methodology

### 2.1. Data

We used the descriptive-analytical method based on a description of the study phenomenon. Its purpose is to systematically describe the facts and characteristics of a given population or an area of interest. We collected data from the study sample using a questionnaire.

**Figure 1:**
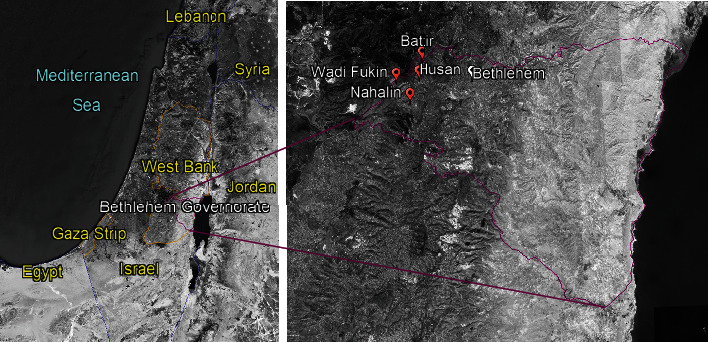
Location of the Bethlehem Governorate and the villages included in this study (Source, Google Earth [56], UN OCHA [57], and Sandvik [58]).

We selected four villages to form western Bethlehem Governorate: Battir, Husan, Nahhalin, and Wadi Fukin, with 4,861, 7,295, 9,047, and 1,389 inhabitants, respectively, in 2019 ([54], Figure 1). We selected these villages for having the most robust agricultural activity in Bethlehem [55]. This is due to the availability of water springs and suitable land. None of them have a proper drainage system or sewer network, discharging sewage in cesspits. Only Nahhalin has a small WWTP serving about 8% of the village. The other towns have seven onsite treatments, each one serving one house. A high percentage of households separates greywater and uses it without further treatment in garden irrigation, especially in the summer.

**Figure 2:**
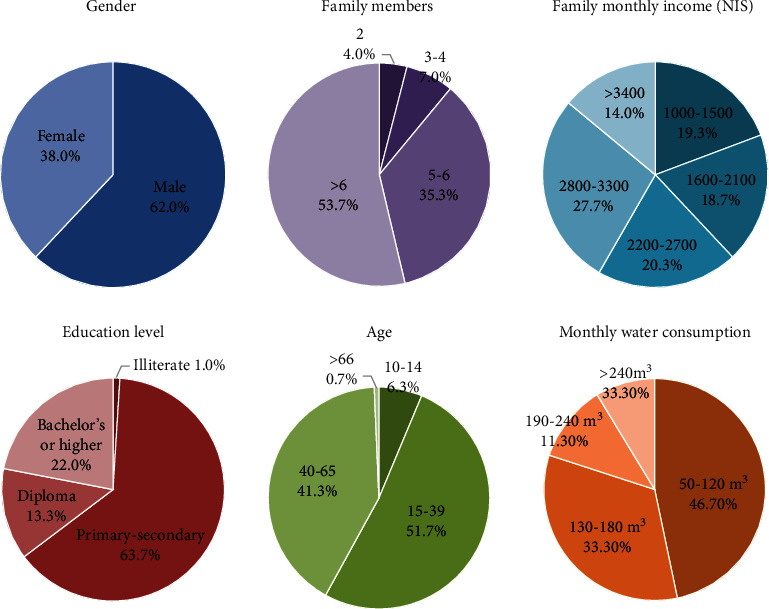
Demographics and water use habits of the sample.

We took a sample of 378 people from the local population concerning residence, gender, education level, age category, number of family members, average monthly income, and average monthly water bill. The demographics of the sample can be seen in Figure 2. Since the interviewed person was always the individual responsible for the agricultural activities in the house, the sample is predominantly male, as farming is traditionally a masculine activity in Palestine. The average interviewee has a primary or secondary education, and their age is between 15 and 65. Also, they live in a household of more than five members where the sample differs from the national average is in the socioeconomic level. Interviewees were distributed more or less evenly according to household income. According to our sample, the average household in the area uses less than 180m^3^/month, much higher than the 30m^3^/month of the Palestinian city of Al-Bireh [59]. For a household of 6.56 members—as it is the average of our sample—these amount to 914 l/capita/day, a much higher value than that Amman (Jordan) 85-100 l/capita/day of Germany 121 l/capita/day, the US 337 l/capita/day [60, 61]. However, it needs to be said in our area of study, most of the water is used for irrigation, and in the references, no agricultural irrigation water is included.

### 2.2. Data Analysis

We used a semistructured questionnaire to collect data from the study sample. It consisted of two major parts: first included general information about the respondents—demographic variables and wastewater practices. The second part focused on the acceptance of greywater reuse. The latter was divided into three sections, depending on the treatment given to greywater: central treatment plant, individual home treatment, and no-treatment. Each section included the same ten potential uses, which had to be ranked between 1 (strongly against) and 5 (strongly in favor).

We estimated the sample using a sample calculator [62] with a margin of error of 5% and a confidence level of 95%. We distributed 378 questionnaires among the villages, as shown in Table 1. 326 were returned, including 26 invalid ones we removed from the analysis as they were incomplete.

The validity of the questionnaire was verified after it was presented in its primary form to several arbitrators who are specialists in the field of water and environmental engineering. They made their suggestions and remarks on the questionnaire. Then, it was redesigned in its final version considering their views.

We used a Likert scale to analyze raw data to replace verbal answers with digital ones, ranging from 1, “strongly disagree” to 5, “strongly agree.” Chronbach-Alpha test was used to determine the reliability coefficient. Descriptive statistics such as frequencies and means were used in the analysis. We tested our hypotheses were using a *t*-test and one way-ANOVA test using SPSS (version 21).

## 3. Results and Discussion

### 3.1. Agricultural and Wastewater Practices

Table 2 shows that a majority of the household have a farm or garden near the house. 60% of them irrigate it with municipal water. For the same purpose, 31.7% of households use raw greywater as the primary source, while 4.6% use it combined with other sources like wells or municipal water, and 3.3% of them use all these three sources together. 91% of households have cesspits, most of which infiltrate wastewater into aquifers. 47.7% of households do not douche their cesspit tank, and 52.3% do it only once a month. Several interviewees mentioned the high price of water as a reason for not doing it more often. 54.7% of cesspits cause unpleasant smells, leakage to neighboring properties, or both. 11% of households had someone with skin or gastrointestinal diseases that could be linked to raw wastewater. Other diseases include bacterial such as salmonellosis, shigellosis, diarrhea, trachoma, and melioidosis; viral: hepatitis A; and diseases caused by parasites: giardiasis, dwarf tapeworm infection, threadworm infection, and hookworm infection. About half of the interviewees know greywater is the most significant fraction of domestic wastewater, and a similar percentage separates it from black water. Although 31.7% of households use greywater, most do not treat it despite being aware of its adverse effects on plants and soils.

### 3.2. Reuse Acceptance without Treatment

Untreated greywater is collected in a pool, from which it eventually infiltrates the soil and mixes with groundwater. Its only supported use is bush irrigation (e.g., garden type), with 62.6% acceptance. Other options like crop irrigation, stone cutting, construction, and public toilet flushing received only mild support. Responders did not support the reuse of greywater of untreated for direct contact applications—aquaculture (24.4% support), aquifer recharge (12.4%), olive pressing (11.0%), and drinking (7.0%) (Table 3).

Abu Madi et al. [52] also assessed the acceptance of greywater reuse in rural Ramallah and Al-Bireh Governorate. As in western Bethlehem, reuse for bush irrigation had very high approval (96%). Other uses received far more support, including stone cutting (97%), car washing (98%), and crop irrigation 89%. Direct contact applications showed lower acceptance again, but still above ours. As for centralized treatment, these differences were noticeable for aquifer recharge and aquaculture—32% and 44%—but far more significant for olive pressing (53%).

### 3.3. Reuse Acceptance after Home Treatment

The acceptance of greywater reuse after home treatment (e.g., onsite treatment plants that offers typical physical treatment with sedimentation) can be seen in Table 4. The results are very similar for those with no treatment. As in that case, the only supported alternative is bush irrigation, with 62.3% acceptance, and direct contact uses are not widely supported. Once more, responses are intermediate for crop irrigation, stone cutting, construction, car washing, and flushing of public toilets. For this treatment alternative, Abu Madi et al. [52] also found high levels of acceptance for crop and bush irrigation, car-washing, and stone cutting, and lower levels for direct contact applications.

### 3.4. Reuse Acceptance after Centralized Treatment

The acceptance levels of using greywater after treatment in a centralized treatment plant (e.g., typical wastewater treatment plants that include physical, chemical, and biological treatments) are summarized in Table 5. Respondents usually supported reuse in crop and bush irrigation—62.3% and 73.6%, respectively. They are undecided about stone cutting, building, car washing, or public toilet flushing. The least accepted uses were those perceived as having contact with people who received less support than after-home treatment: aquifer recharge (17.3%), aquaculture (17.0%), and olive pressing (14.4%).

As in western Bethlehem, reuse for bush irrigation had very high acceptance (80%) in Ramallah [52]. Although reuse for direct contact applications was not highly supported there either, their acceptance was higher than in our case. Aquifer recharge and aquaculture showed a noticeable higher acceptance—38% and 42%, respectively—while olive pressing has roughly 20% more approval (51%).

### 3.5. Treatment-Reuse Acceptability

**Figure 3:**
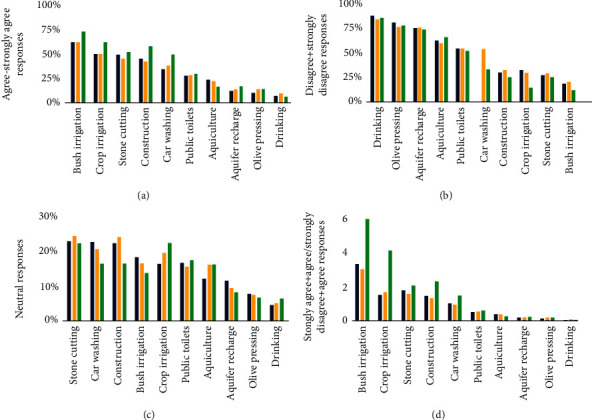
Relative distribution of acceptability for greywater reuse according to treatment and applications: (a) positive responses, (b) negative response, (c) undecided/indifferent responses, and (d) positive/negative ratio.

The relative distribution of acceptability for the different uses of treated greywater according to their treatment is shown in Figure 3. As it can be seen in Figures 3(a) and 3(d), centralized treatment is generally the best-regarded treatment, particularly when it comes to reuses without perceived direct contact: bush and crop irrigation, stone cutting, construction, and car washing. On the other hand, home treatment and no treatment present relatively similar results, predominantly positive and negative responses (Figures 3(a) and 3(b)). This added to the more significant number of neutral responses for home treatment for most reuses (Figure 3(c)) might indicate a general mistrust in the capabilities of home treatment. This lack of self-reliance was even more pronounced in Abu Madi et al. [52], where home treatment was the least supported alternative, while no treatment and centralized treatment present similar acceptance levels. Even in Taher et al. [53], 32.9% of the users who were satisfied with their treatment plant at home would prefer a centralized WWTP if they were connected to the sewer system. It might be possible to suggest then that home treatment is a temporary solution while the sewerage is extended.

While bush irrigation is the only reuse accepted across all treatments (Tables 3–5), Figure 3 shows, in addition, a moderately positive perception of greywater reuse for crop irrigation, stone cutting, and construction—all uses seen as without direct contact with humans. It also highlights a clear rejection of reuses with direct contact: drinking, olive pressing, aquifer recharge, aquaculture, and, to a lesser extent flushing of public toilets. Car washing, relatively new reuse as mentioned in the introduction, falls somewhere in between, with also a high number of respondents being unsure/undecided (Figure 3(c)).

The idea that greywater is appropriate for irrigation but inadequate for other activities is consistent with several studies worldwide [41]. In the nine studies from Australia and the US reviewed by Po et al. [63], garden irrigation was opposed by only 6% of the respondents or less. However, this fraction increased for irrigating hay or alfalfa (8%), orchards (10%), dairy pastures and vineyards (15%), or vegetable crops (21%). In another Australian study, Marks et al. [40] identified a decrease in support of greywater reuse for irrigation when moving from nonagricultural uses—golf courses, parks, gardens (96.6% acceptance), schoolyards, and playing fields (86.6%)—to agricultural land—pastures (76.0%), vineyards (73.9%), and vegetable and fruit crops (68.2%). In this previous study, it was observed that, contrary to rural Bethlehem, urban Australia is far more supportive of reusing greywater for flushing toilets in public buildings—94.2% support—and car washing—91.3% support. Also higher, but closer to our case study, were the acceptance levels of these two uses in the Muscat Governorate of Oman—with 66.3 and 53.3%, respectively [38]. Another commonality between the Omani study and ours was their opposition to drinking reused water, as 81.7% of the respondents did not think greywater could be treated to such an extent. A similar level of distrust was found in Turkey by Buyukkamaci and Alkan [39], although they assessed treated wastewater in general, not greywater specifically.

### 3.6. Demographics and Acceptance

As mentioned in the introduction, Dolinčar and Saunders [36] concluded that the acceptance of recycled water correlates with a high level of education and younger age, while income and gender are not always significant predictors. In our case, however, the level of acceptance of the different reuse alternatives is, in general terms, independent of demographics (Table 6). This is true for family size, income, and monthly water bills. Regarding gender, we observed that women tended to see aquaculture more positively than men, with 2.44 and 2.15, respectively. Regarding age, the only statistically significant difference appeared for aquaculture between respondents of ages 15 to 39 and those between 40 and 65, as the latter supported this reuse—mean of 2.21 vs. 2.53. The level of education affected responses in two different ways. Acceptance of reuse for public toilet flushing tended to decrease with the level of education. We also found that illiterate respondents had a more negative attitude towards reuse in construction than the rest of the sample. This differs from Thaher et al. [53], where the acceptance of reused greywater after home treatment was slightly lower for people with higher education (81.2% vs. 87.7%).

## 4. Conclusions and Recommendations

This study analyzed the social acceptance of current greywater reuses in rural Bethlehem, Palestine. Of the three potential treatments for greywater: centralized wastewater treatment plant, home treatment, and no treatment, the former is the most trusted one, partially because respondees are well aware of the limitations of the other two, having to deal with them—technical problems, bad smells, etc. Regarding potential reuses, we found similarities among the four villages evaluated and concluded that the most accepted reuse option is in bush irrigation since it is perceived not to have any contact with humans. We found out that other uses without direct contact like crop irrigation, stone cutting, and construction received less support but were still seen favorably. Car washing and public toilet flushing received intermediate responses. A possible explanation for this is that they are relatively new applications, and the general public might still be unsure about them. Information campaigns in this direction might help increase the acceptance of these alternatives. Finally, uses perceived as having direct contact with humans, like drinking, olive pressing, aquaculture, and aquifer recharge, were not supported. To popularize these and other alternatives, we need to raise awareness that different uses have different quality requirements, and if the water fulfills them, its use is safe.

## Figures and Tables

**Table 1:** Distribution of study sample among villages.

Village	Population	Sample members
Nahhalin	9,047	117
Husan	7,295	95
Battir	4,861	68
Wadi Fukin	1,389	20
Total	22,592	300

**Table 2:** Agricultural and wastewater practices of the sample.

Variables	Variables values	Frequency	Percentage
Is there a farm/garden near the house?	Yes	208	69.3%
	No	92	30.7%

How large is the agricultural area?	50-100 m^2^	93	31.0%
	120-170 m^2^	44	14.7%
	180-230 m^2^	22	7.3%
	240 m^2^ or more	49	16.3%

What water sources are available for agriculture?	Municipal water	179	59.7%
	Hose well	49	16.3%
	Spring	23	7.7%
	Greywater	16	5.3%
	Municipal water and hose well	19	6.3%
	Municipal and greywater	4	1.3%
	Municipal, greywater, and hose well	10	3.3%

Is there a cesspit at your house?	Yes	272	90.7%
	No	28	9.3%

What is the type of cesspit tank?	Infiltration	183	61.0%
	No infiltration	117	39.0%

How often do you douche a cesspit tank?	Every 15 days	31	10.3%
	Every 30 days	41	13.7%
	Every 60 days	85	28.3%
	Never	143	47.7%

Do you have or had problems with the cesspit tank?	Unpleasant smells	66	22.0%
	Leak to neighbors	16	5.3%
	Both	82	27.3%
	No problems	136	45.3%

Are there any diseases in your family that could be linked to raw wastewater?	Skin diseases	24	8.0%
	Gastrointestinal disease	9	3.0%
	Other diseases	122	40.7%
	No diseases	145	48.3%

Does your plumbing separate grey and black water?	Yes	155	51.7%
	No	145	48.3%

Did you know that 80% of sewage is made up of greywater?	Yes	166	55.3%
	No	134	44.7%

Is the raw greywater used in irrigation around the house?	Yes	95	31.7%
	No	205	68.3%

Did you treat greywater before using it in irrigation?	Yes	46	15.3%
	No	254	84.7%

Did you know that irrigating with raw greywater harms soil and plants?	Yes	223	74.3%
	No	77	25.7%

**Table 3:** Reuse acceptance without treatment.

Usage field	Strongly disagree	Disagree	Neutral	Agree	Strongly agree	Mean	Answer
Bush irrigation	24	32	56	133	55	3.54	Agree
	8.0%	10.7%	18.7%	44.3%	18.3%		
Crop irrigation	40	59	50	121	30	3.14	Neutral
	13.3%	19.7%	16.7%	40.3%	10.0%		
Stone cutting	33	49	70	117	31	3.21	Neutral
	11.0%	16.3%	23.3%	39.0%	10.3%		
Construction	34	61	68	103	34	3.14	Neutral
	11.3%	20.3%	22.7%	34.0%	11.3%		
Car washing	33	81	69	90	27	2.99	Neutral
	11.0%	27.0%	23.0%	30.0%	9.0%		
Public toilets	57	108	51	62	22	2.61	Neutral
	19.0%	36.0%	17.0%	20.7%	7.3%		
Aquaculture	79	111	37	62	11	2.38	Disagree
	26.3%	37.0%	12.3%	20.7%	3.7%		
Aquifer recharge	124	104	35	29	8	1.98	Disagree
	41.3%	34.7%	11.7%	9.7%	2.7%		
Olive pressing	139	104	24	30	3	1.85	Disagree
	46.3%	34.7%	8.0%	10.0%	1.0%		
Drinking	161	104	14	11	10	1.68	Disagree
	53.7%	34.7%	4.7%	3.7%	3.3%		

**Table 4:** Reuse acceptance after home treatment.

Usage field	Strongly disagree	Disagree	Neutral	Agree	Strongly agree	Mean	Answer
Bush irrigation	23	39	51	129	58	3.53	Agree
	7.7%	13.0%	17.0%	43.0%	19.3%		
Agriculture use	43	47	59	112	39	3.19	Neutral
	14.3%	15.7%	19.7%	37.3%	13.0%		
Stone cutting	25	64	74	107	33	3.19	Neutral
	8.3%	21.3%	24.7%	34.7%	11.0%		
Construction	27	72	73	95	33	3.12	Neutral
	9.0%	24.0%	24.3%	31.7%	11.0%		
Car washing	37	84	63	89	27	2.95	Neutral
	12.3%	28.0%	21.0%	29.7%	9.0%		
Public toilets	64	102	47	66	21	2.59	Neutral
	21.3%	34.0%	15.7%	22.0%	7.0%		
Aquaculture	91	91	49	54	15	2.37	Disagree
	30.3%	30.3%	16.3%	18.0%	5.0%		
Aquifer recharge	130	99	29	29	13	1.99	Disagree
	43.3%	33.0%	9.7%	9.7%	4.3%		
Olive pressing	138	94	23	39	6	1.94	Disagree
	46.0%	31.3%	7.7%	13.0%	2.0%		
Drinking	169	85	16	20	10	1.72	Disagree
	56.3%	28.3%	5.3%	6.7%	3.3%		

**Table 5:** Reuse acceptability after centralized treatment.

Usage field	Strongly disagree	Disagree	Neutral	Agree	Strongly agree	Mean	Answer
Bush irrigation	12	25	42	145	76	3.83	Agree
	4.0%	8.30%	14.00%	48.3%	25.3%		
Crop irrigation	17	28	68	126	61	3.62	Agree
	5.7%	9.30%	22.7%	42.0%	20.3%		
Stone cutting	30	46	68	99	57	3.36	Neutral
	10.0%	15.30%	22.7%	33.0%	19.0%		
Construction	22	54	50	125	49	3.42	Neutral
	7.3%	18.00%	16.7%	41.7%	16.3%		
Car washing	33	68	50	112	37	3.17	Neutral
	11.0%	22.70%	16.7%	37.3%	12.3%		
Public toilets	56	100	53	59	32	2.7	Neutral
	18.7%	33.30%	17.7%	19.7%	10.7%		
Aquaculture	82	118	49	42	9	2.26	Disagree
	27.3%	39.30%	16.3%	14.0%	3.0%		
Aquifer recharge	120	103	25	48	4	2.04	Disagree
	40.0%	34.30%	8.3%	16.0%	1.3%		
Olive pressing	147	89	21	32	11	1.9	Disagree
	49.0%	29.7%	7.0%	10.7%	3.7%		
Drinking	180	80	20	15	5	1.62	Disagree
	60.0%	26.7%	6.7%	5.0%	1.7%		

**Table 6:** Multivariate correlations between demographic variables and acceptance of greywater reuse.


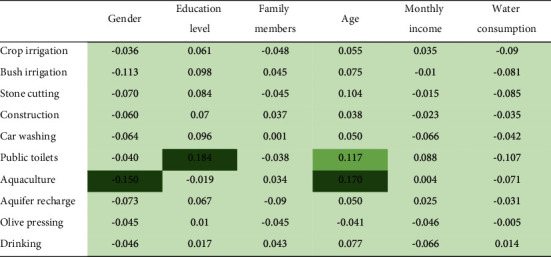

## Data Availability

The data used to support the findings of this study are available upon request from the corresponding author.
